# Methods and Strategies for Reducing Seclusion and Restraint in Child and Adolescent Psychiatric Inpatient Care

**DOI:** 10.1007/s11126-021-09887-x

**Published:** 2021-02-25

**Authors:** Charlotta Perers, Beata Bäckström, Björn Axel Johansson, Olof Rask

**Affiliations:** 1 Skåne Child & Adolescent Psychiatry, Malmö, Sweden; 2 Skåne Child & Adolescent Psychiatry, Unit for Pediatric Bipolar & Psychotic Disorders, Lund, Sweden; 3 Department of Clinical Sciences Lund, Division of Child & Adolescent Psychiatry, Lund University, Lund, Sweden; 4 Skåne Child & Adolescent Psychiatry, Regional Inpatient Care, Emergency Unit, Malmö, Sweden

**Keywords:** Restraint, Seclusion, Child, Adolescent, Inpatient, Psychiatry

## Abstract

Restraints and seclusions are restrictive interventions used in psychiatric inpatient units when there is an imminent risk of harm to the patient or others. Coercive measures are controversial and can lead to negative consequences, including negative emotions, re-traumatization, injuries, or death. The article summarizes the last 10 years of literature regarding methods and strategies used for reducing seclusions and restraints in child and adolescent psychiatric inpatient units, and reports on their outcomes. The literature was reviewed by searching PubMed and PsycInfo for English-language articles published between May 2010 and May 2020. Eighteen articles were found that described methods or strategies aimed at reducing restraint or seclusion utilization in child and adolescent psychiatric inpatient units. The following interventions were evaluated: Trauma-Informed Care (TIC), Six Core Strategies, Child and Family Centered Care (CFCC), Collaborative & Proactive Solutions (CPS), Strength-Based Care, Modified Positive Behavioral Interventions and Supports (M-PBIS), Behavioral Modification Program (BMP), Autism Spectrum Disorder Care Pathway (ASD-CP), Dialectical Behavior Therapy (DBT), sensory rooms, Mindfulness-Based Stress Reduction Training (MBSR) of staff, and Milieu Nurse-Client Shift Assignments. Most of the interventions reduced the use of seclusions and/or restraints. Two child-centered and trauma-informed initiatives eliminated the use of mechanical restraints. This review shows that the use of coercive measures can be reduced and should be prioritized. Successful implementation requires ongoing commitment on all levels of an organization and a willingness to learn. To facilitate comparisons, future models should evaluate different standardized parameters.

## Introduction

Restraints and seclusions are restrictive interventions used in psychiatric inpatient units, including units for children and adolescents, when there is an imminent risk of harm to the patient or others [1]. These methods are controversial. Restraints have been associated with many adverse effects, and put both patients and staff at risk of injury and death [2, 3]. In 1998, reports in the Hartford Courant revealed that 142 patients in the U.S. had died in the previous 10 years as a result of restraint [4]. Many of these were children who had died of asphyxiation [5]. A recent systematic review on the effects of coercive interventions in adult psychiatry estimated an incidence of post-traumatic stress disorder after seclusion or restraint varying from 25 to 47% [6]. Other consequences were revival of previous traumatism, hallucinations, and negative emotions, particularly feelings of punishment and distress. Restraint reduction can lead to reduced costs, reduced sick time among staff, fewer injuries to adolescents and staff, and reduced staff turnover [7].

The definition of restraint varies between countries, but refers broadly to an involuntary restriction of movement through manual holds, mechanical devices, or medication. Mechanical restraint involves some form of restrictive device, such as belts or a specially designed bed [2]. Chemical or pharmacological restraint refers to an intramuscular injection of a sedative medication. Seclusion is defined as an involuntary confinement of a patient in a room where the patient is physically prevented from leaving.

Legislation regarding coercive measures differs between countries. There is a movement towards less coercion and, as attitudes change, legislation revision follows. As an example, The Compulsory Mental Care Act in Sweden was recently updated, reducing the maximum duration of bed restraints for children from 4 h to 1 h and seclusions from 8 to 2 h [1]. This development is in alignment with the United Nations Convention on the Rights of the Child, which states that, in all actions concerning children, the best interests of the child is to be a primary consideration [8].

Mechanical restraints are probably the least accepted containment measure, and have been described as distressing and inhumane by Finnish adolescents [9]. In an American study, children and adolescents aged 12–15 reported that they associated fear, anger, and re-traumatization with the use of physical restraint [10]. Adult patients and staff in emergency care mental health services in England also reported strong disapproval of any form of mechanical restraint [11]. Seclusion seems to be slightly more accepted among adolescents, when compared to mechanical restraints [9, 12]. However, a systematic review covering adult psychiatric inpatients’ experience of seclusion revealed that, during seclusion, patients felt vulnerable, neglected, and abused, disconnected from the experience, and felt it was dangerous to their mental health [13].

The experience of seclusions and restraints varies among individuals. A systematic review on the effects in adult psychiatry reported that some patients described feelings of safety, help, and clinical improvement, or evaluated the intervention as necessary [6]. Nevertheless, the review concluded that the interventions were mostly associated with negative emotions, particularly feelings of punishment and distress. Therapeutic interaction seemed to influence the perceptions of coercion and reduce negative effects when coercive measures were not avoidable. Similar results were found in a review on adults’ perspectives of being physically restrained. Although some patients described feelings of safety and being helped, and experiencing concern from the nurses while being restrained, they still reported more negative feelings overall [14]. Common themes included anger, fear, humiliation, and powerlessness.

In interviews with psychiatric nurses in Ireland, restraint and seclusion were interpreted as a last resort in the management of client’s aggression and violence [15]. The nurses experienced significant emotional distress when compelled to engage in the interventions and, as a way to get through the incidents, the nurses appeared to suppress their distressing emotions. The harsh nature of these interventions also conflicted with the caring aspects of the nursing role. In a recent systematic review, nurses viewed coercive measures as undesirable but necessary to maintain safety on psychiatric wards [16]. They also expressed a need for less intrusive interventions.

A high proportion of psychiatric patients have a history of trauma. In one review, 90% of people seeking treatment for a variety of psychiatric conditions had been exposed to significant emotional, physical and/or sexual abuse in childhood [17]. Patients with a history of trauma can experience re-traumatization during coercive interventions [6, 14]. Adult patients report that experiences of being restrained brought back memories of previous trauma, including rape and child abuse [14]. Trauma has also been suggested to increase the risk of being secluded or restrained. In a study from Iowa (2011), 70% of the adult inpatients who experienced the most instances of seclusions or restraints had experienced childhood abuse [18]. Another study found that children with post-traumatic stress disorder (PTSD) and/or a history of physical abuse had a significantly higher risk of being secluded or restrained in a U.S. pediatric day hospital [19].

In a previous review, Valenkamp et al. (2014) searched for empirical studies that aimed to reduce restraints/seclusions in child and adolescent psychiatric inpatient units, with a pre-post design between 2006 and 2013 [20]. Three articles met their inclusion criteria. Two of the studies evaluated Collaborative & Proactive Solutions, formerly Collaborative Problem Solving (CPS), Ross Greene’s cognitive-behavioral approach for working with aggressive children [21, 22]. The third study covered a milieu-based comprehensive behavioral management program, where the interventions aimed at changing patient behavior [23]. All studies reported reduction in the number of restraints.

The prevalence of seclusion and restraint use between 2000 and 2010 in child and adolescent psychiatric settings was examined in a systematic review by De Hert et al. (2011) [24]. The baseline rates of seclusions were 26% of patients or 67 per 1000 patient days, and the baseline rates of restraints were 29% of patients or 42.7 per 1000 patient days. Recent studies on child and adolescent psychiatric inpatient units reported rates of restraint between 6.5 and 29% of admitted patients; 6.5% restraint, Norway [25, 26], 12% restraint or seclusion, the U.S. [19], 26.9% restraint or seclusion, Australia [27], 29% restraint or seclusion, the U.S. [28], 16.9% restraint or seclusion, the U.S. [29], 6.9% mechanical restraint only, Finland [30]. A review by Beghi et al. (2013) reported a 3.8–20% prevalence of restraint utilization in adult psychiatric units [31].

The purpose of this review was to summarize the last 10 years of literature regarding methods and strategies currently used for reducing seclusions and restraints in child and adolescent psychiatric inpatient units.

## Methods

This review began with a search of PubMed and PsycInfo for papers in English published between May 15th 2010 and May 15th 2020, searching for the following key words in the title/abstract or as MeSH-terms: “(Restraint OR chemical restraint OR pharmacological restraint OR physical restraint OR mechanical restraint OR seclusion OR restraint MeSH) AND (Child OR adolescent OR child MeSH OR adolescent MeSH) AND (Inpatient OR inpatient MeSH) AND (Psychiatry OR psychiatric OR mental OR adolescent psychiatry MeSH OR child psychiatry MeSH)”. The inclusion criteria were published articles that described methods or strategies aimed at reducing restraint or seclusion utilization in child and adolescent psychiatric inpatient units, with full text articles in English. The exclusion criteria were unpublished material, articles focusing on adult psychiatric units, and articles about other settings than child and adolescent psychiatric inpatient units, e.g. residential facilities. We also excluded articles that focused solely on pharmacological approaches to aggressive behavior. Using an integrative approach, studies were not otherwise excluded due to methodology (e.g. experimental or non-experimental).

We initially considered 73 abstracts in PsycInfo and 79 papers in PubMed. There was a fairly large overlap and many articles focused on adults only. Thirteen articles were selected for our review. We also found five additional studies that met our inclusion criteria, through scrutinizing the reference lists of other articles, resulting in a total of 18 papers in the review. Four of the articles described the same two facilities but were included since the evaluations in the studies were made months/years apart [32–35]. Data extracted from the articles included type of interventions, study design, setting, context, and outcome. The studies were then categorized according to identified themes.

## Results

The 18 articles are described in Table 1. Studies were conducted in the U.S. (*n* = 15), Australia (*n* = 1), New Zealand (*n* = 1), and Canada (*n* = 1). The reviewed articles displayed a wide range of study designs, interventions, and outcome measures, and could be divided into six themes or groups.

**Table 1:** Summary of studies included in the present review

Author (year)	Intervention – Group 1	Design	Setting	Context	Outcome
Azeem et al. (2015) [33]	Trauma-informed, strength-based care. Focus on primary prevention. The interventions aligned with the Six Core Strategies: Leadership towards org change Use of data to inform practice Workforce development R/S reduction tools Patient and family inclusion Debriefing	Descriptive article of a 10-year quality improvement project 2005–2014. Pre-post measures. Total number of admissions: 178 (2005), 163 (2014).	U.S. 52-bed Pediatric Psychiatric Hospital. Four units.	Youths usually presented with severe aggression and/or self-injurious behaviors, who often required multiple restraints (physical and/or mechanical) at other inpatient settings prior to admission. A large number had experienced repetitive trauma.	*Statistical significance not mentioned* 100% decrease in mechanical restraints: - 485 in 2005 - 0 in 2014 88% decrease in physical restraints: - 3033 in 2005 - 379 in 2014
Azeem et al. (2011) [32]	A previous evaluation of the Six Core Strategies at the same facility.	Retrospective. Based on about 33 months data collection. Pre-post measures. 458 youths admitted between July 2004–March 2007. Training in Six Core Strategies began March 2005.	U.S. 26-bed child and adolescent unit divided into three units. Patients 6–17 years old.	Patients with disruptive behavior disorders, mood disorders, reactive attachment disorder, substance abuse, pervasive developmental disorder, mental retardation.	*Statistical significance not mentioned* Pre Six Core Strategies: 93 S/R - 73 seclusions - 20 restraints Post Six Core Strategies: 31 S/R - 6 seclusions - 25 restraints
Wisdom et al. (2015) [36]	Six Core Strategies: Leadership towards org change. Use of data to inform practice. Workforce development. S/R reduction tools. Patient and family inclusion. Debriefing. Based on trauma-informed care.	Descriptive article of the implementation of Six Core Strategies in three children’s mental health facilities 2007–2011. Pre-post measures.	U.S. 1. Children’s psychiatric center 2. Children’s residential treatment facility 3. Private psychiatric hospital for children and adolescents. Patients up to 17 years old.	High utilization of restrictive interventions. Majority of patients with ADHD, conduct disorders or mood disorders.	Statistically significant decrease in use of S/R in all three facilities: 1. 62% (from 67 to 25 S/R per 1000 client days, *p* = .019) 2. 86% (from 63 to 7 S/R per 1000 client days, *p* = .001) 3. 69% (from 99 to 13 S/R per 1000 client days, *p* = .007)
Author (year)	Intervention – Group 2	Design	Setting	Context	Outcome
Regan et al. (2017) [37]	CFCC including CPS and trauma-informed approach. CFCC is a philosophy of care based on treating people with dignity and respect, sharing information with patients/families, and supporting them to build on their strengths and independence and to collaborate in policy/program development.	Descriptive article of an inpatient unit that implemented the principles of CFCC, including CPS and a trauma-informed approach. Pre-post measures.	U.S. 13-bed inpatient child psychiatric unit. Patients 2–12 years old.	Patients with PTSD, anxiety disorders, ADHD, mood disorders, learning disorder, psychotic disorders, pervasive mental developmental disorders. Pre CFCC: The unit used a behaviorally oriented level system where children earned freedom and privileges based on their ability to conform to the rules. Misbehavior led to time-outs and high levels of S/R (twice the state average for child units). Staff unhappy, felt unappreciated.	No mechanical restraints since 2001. No locked-door seclusions or chemical restraints since 2002. 2003 reduction in brief physical holds (under 5 min) from over 100 to 10 or less/months. Significant decrease in staff injuries (P value not mentioned). Reduced staff-turnover. Increased job satisfaction.
Ercole-Fricke et al. (2016) [38]	CPS: Cognitive behavioral approach focused on adult-child decision making. Conceptualizes maladaptive behaviors as the byproduct of lagging cognitive skills. The CPS was modified to suit the population and the “short-stay” environment.	Quantitative, comparative, quasi-experimental design. Retrospective. Compare behavioral outcomes and staff perceptions. Pre-post design including all discharged patients 2008 (pre) and 2012 (post)	U.S. Adolescent acute psychiatric inpatient unit. Patients 12–17 years old.	Pre CPS: The unit used a behavioral model where misbehavior entailed point reduction and loss of privileges, time-outs, or open seclusions.	Statistically significant decrease in: - Punitive strategies and techniques (open-door seclusion decreased from 61 events in 2008 to 0 events in 2012) (*p* = .001) - Behaviors related to the need for restraints and self-inflicted injury (*p* = .005) - Need for security staff involvement (*p* = .001) No significant decrease in restraint episodes but continued to trend downward: 25 restraint episodes 2008, vs 17 in 2012.
Sams et al. (2016) [39]	Strength-Based Care Exploring patients’ goals, strengths, relationships, skills, and family communication. Different interventions: - CPS - Mindfulness groups - DBT group - Animal assisted therapy - ACT - Narrative therapy - Family Movie Therapy	Descriptive article of an inpatient unit’s integration of a strength-based approach with a traditional, medical model of psychiatric care. The unit implemented CPS in 2006.	U.S. 24-bed acute child and adolescent psychiatric inpatient unit. Patients 5–18 years old.	Pre strength-based care: The unit used a behavioral approach (rewards and consequences based on patients’ behavior) and medication management.	*Statistical significance not mentioned.* 75% reduction in total hours of seclusion and restraint noted over the course of 1-year following the implementation of the strength-based program revisions. Increase in patient, family, and staff satisfaction.
Weldon Bonnell et al. (2014) [40]	External reviewers assessed the unit June 2009 ➔ - Restructuring unit staffing - Regular debriefing sessions - Staff learning CPS - Broad representation of multidisciplinary team members	Retrospective. Pre-post quality assurance assessment, comparing data from Jan 2008-Dec 2009 (pre) to data from Jan-Dec 2010 (post). Admitted patients: 85 in 2008–2009 (pre) 39 in 2010 (post)	Canada. 7-bed child and adolescent inpatient psychiatric facility. Mean patient age 14–15 years.	Majority of patients with mood disorders, ADHD, adjustment disorder, anxiety disorder, psychotic disorder, substance-related disorder. 2008–2009 marked escalation in physical aggression, constant observation, security, staff sick leave.	Statistically significant decrease in constant observation (*p* = .002). Not statistically significant decrease in seclusion: Mean number of min patients spent in seclusion each month decreased from 49.8 (pre) to 16.7 min (post). Physical restraints were not used at all during the study period. Not statistically significant decrease in incidents (verbal/physical aggression, errors by staff, accidents) and security.
Author (year)	Intervention – Group 3	Design	Setting	Context	Outcome
Reynolds (2016) [41]	M-PBIS: 1. Universal prevention practices. Positively worded behavioral expectations. Rewards for positive behavior. 5:1 positive-negative adult-child interaction ratio. Feedback to staff. 2. Targeted interventions 3. Intensive individualized interventions	Naturalistic, prospective 4-year study Jan 2010-June 2014. 726 admissions (pre) 759 admissions (post)	U.S. 12-bed high-risk youth psychiatric inpatient unit. Mean patient age 13.18 years.	Acute care for youth in crisis, typically involving threats to harm self or others.	S/R events decreased from 543 to 253. Significant decrease in: - Mean seclusion rate (from 1.49 to 0.73 per 1000 patient hours, *p* = .02) - Percentage of patients placed in S/R (from 19.6% to 13.4%, *p* = .001) - Mean duration of S/R (from 20.43 to 8.18 min/episode, *p* = .001) - Percentage of patients who received PRN for agitation (from 42% to 30%, *p* = .001) Seclusions included both open-door and locked-door seclusions
Carlson et al. (2020) [42]	Pre: BMP with behavioral classroom management, parent training, point system, social reinforcement, time-out. Post: BMP-absent – “No comprehensive treatment approach”. Verbal de-escalation or distraction, teaching coping skills, point system, no limit setting.	Retrospective cohort study. 5 cohorts over 10 years 2008–2018. Total of 661 admissions from the 5 cohorts. Evaluated PRN for agitation and S/R/H.	U.S. 10-bed children’s inpatient unit. Patients 5–12 years old.	77% of the patients admitted for aggression. High rates of ADHD, ODD.	BMP-absent children had significantly higher rates of: - S/R/H (mean value increased from 17 to 65 per 1000 client days, *p* < .001). - PRN use (mean value increased from 163 to 483 per 1000 client-days) (*p* < .001)
Eblin (2019) [43]	Decision-making algorithm for initiation of S/R. Behavioral modification plans for patients at risk for S/R. Patient debriefing after S/R.	Quality improvement study. Post-implementation, data collection 3 months Sept-Nov 2018.	U.S. 14-bed inpatient child and adolescent behavioral health unit. Patients 6–17 years old.	Patients with mood disorders, anxiety disorders, neurodevelopmental and eating disorders, trauma, disruptive disorders, impulse-control, and conduct disorders.	*Statistical significance not mentioned.* 55% decrease in total S/R rates (from 0.031 to 0.0137 per 1000 client days) (62% decrease in seclusions, 18% decrease in restraints, 29% decline in mean duration of time spent in S/R from 69 to 49 min per episode)
Author (year)	Intervention – Group 4	Design	Setting	Context	Outcome
Seckman et al. (2017) [44]	Sensory room 1:1 patient-staff intervention. Staff training included in the intervention.	Plan-do-check-act (PDCA) model with pre-post measures. S/R rate measured 6 months pre and 6 months post sensory room initiation.	U.S. 20-bed adolescent psychiatric inpatient unit. Patients 12–17 years old.	Patients with emotional and behavioral disorders such as mood disorders, adjustment disorders, psychotic disorders, conduct or oppositional defiant disorders, PTSD, autism.	*Statistical significance not mentioned.* 26.5% reduction in restraint and 32.8% reduction in seclusion incidents. 16.4% frequency reduction in patient aggression. Increase in seclusion duration by 17%, and restraint duration by 31%.
West et al. (2017) [45]	Sensory room 1:1 patient-staff intervention. Staff training included in the intervention.	Open trial. Pre-post design. Retrospective review of medical files. Evaluation period June 2011-Oct 2012. Sample: 56 sensory room users and 56 non-users.	Australia. 20-bed adolescent psychiatric inpatient unit. Patients 12–18 years old.	Patients with mood or anxiety disorders, trauma, personality disorders, psychotic or disruptive disorders, neurodevelopmental disorders, substance-related and eating disorders.	Statistically significant distress reduction following sensory room use (*p* < .001) Greatest distress reduction among adolescents with a history of aggression (*p* < .01) No significant difference in seclusion rates.
Bobier et al. (2015) [46]	Sensory room 1:1 patient-staff intervention. Staff training included in the intervention.	Pilot investigation. Evaluation 6 months before, during (Nov 2012-May 2013) and 6 months after introduction of sensory room.	New Zealand. 16-bed specialist mental health facility. Patients up to 18 years old.	Patients with mood or anxiety disorders, trauma, psychotic disorders, disruptive or neurodevelopmental disorders, who could not be treated or managed in other mental health care settings. The unit provided intensive treatment and specialist assessment.	Statistically significant decrease in seclusion episodes (from 73 to 26 or 3.2 to 1.8 per 100 treatment days, *p* < .001) Full restraint decreased slightly. Statistically significant increase in partial restraints (from 18 to 44 *p* < .001) (Full vs partial restraint not defined.)
Hallman et al. (2014) [47]	Brief (8 days) MBSR-training of staff: - Waking up from autopilot - Body scanning - Refraining from judgement - Breathing as a stress reliever - Befriending yourself	One-group repeated measure design. Evaluation before MBSR training and 2 months later. 12 participants completed the MBSR. Scales used for evaluation: - Perceived Stress Scale - Toronto Mindfulness Scale	U.S. 14-bed child and adolescent psychiatric acute care unit.	High level of patient acuity. Typical diagnoses included major depression with suicidal ideation, psychosis, and autism spectrum disorders, among others.	Statistically significant stress reduction and increased mindfulness (*p* < .05) among participating staff post-MBSR. Not statistically significant decrease in S/R episodes (from 30 to 10), decrease in 1:1 (209 to 183) and 2:1 (46 to 0) staff per patient episodes.
Rae Magnowski and Cleveland (2020) [48]	Change in nurse staffing structures: One “milieu nurse” responsible for providing structure, safety, early identification of crisis, consistency, empathy, PRNs, updating client treatment plans, focus on individual client needs. Combining *cognitive milieu therapy* and *nurse presence*. No additional resources/costs.	Quantitative, retrospective, comparative project design. Sample: All admitted patients (758) between Jan-May 2016 and Jan-May 2017 who were physically or mechanically restrained (57).	U.S. 20-bed child and adolescent psychiatric inpatient unit. Patients 5–18 years old.	Patients with anxiety or mood disorders, obsessive-compulsive or behavioral disorders, among others. Pre milieu nurse: Average monthly restraint rate: 78.4 per 1000 client days 2015, 54.2 per 1000 client days Jan-Sept 2016. High restraint use lead to frequent staff injuries, restraint recidivism and staff turnover.	Statistically significant decrease in average monthly restraint rate: 72.9 (median 61.2) per 1000 client days during control variable, vs 7.5 (median 6.8) per 1000 client days during the intervention (*p* = .004).
Author (year)	Intervention – Group 5	Design	Setting	Context	Outcome
Kuriakose et al. (2018) [35] Cervantes et al. (2019) [34]	ASD-CP - Input from caregivers (communication skills, early warning signs of agitation, activity preferences) - Structured schedule, visual support - Teaching and reinforcing patient coping skills - Training staff in features of ASD Evaluated the stability of the ASD-CP study outcomes (described above)	Pre-post design. Data collected Jan 2014-June 2015 and July 2015-Dec 2016. Sample included 74 children with ASD, low language level and/or high disruptive behavior to the degree that patient needed 1:1 staffing. First time admissions. 18-month follow-up Jan 2017-June 2018 compared to the 18-month initial evaluation and 18-month pre-ASD-CP.	U.S. Pediatric psychiatric emergency unit with a brief stabilization unit and 3 inpatient units with a total of 45 beds. Patients 4–17 years old.		Significantly smaller proportion of children experiencing a hold/restraint after the implementation (pre-ASD-CP 38.8%, post ASD-CP 26.3%, *p* = .039) Not statistically significant, but clinically relevant decrease in the number of holds/restraints (77%), number of intramuscular injections. Significant (*p* < .05) decrease in: -Total number of holds/restraints across settings - Total intramuscular medication administration - Proportion of children experiencing any hold/restraint.
Author (year)	Intervention – Group 6	Design	Setting	Context	Outcome
Tebbett-Mock et al. (2020) [49]	DBT - DBT coaching - Token economy - Chain and solution analyses for egregious behaviors - Therapeutic environment, resources for coping skills - DBT vocabulary - DBT skills groups - Individual and family psychotherapy - Psychoeducation - Staff training	Retrospective chart review for adolescents receiving inpatient DBT (*n* = 425) and a historical control group treated on the same unit before DBT (*n* = 376), receiving treatment as usual (the same seasonal span the year before).	U.S. Coeducational, acute-care inpatient unit within a private psychiatric hospital. Patients 12–17 years old.	Patients with mood disorders, schizophrenia spectrum disorders, anxiety disorders, trauma and stress-related disorders, disruptive disorders, and ADHD, among others. Patients admitted because of imminent safety concerns, including danger to self or others.	Statistically significant decrease in DBT-group vs TAU-group: Number of restraints (Mean 0.14 vs 0.16, *p* = .01) Not statistically significant decline in number of seclusions.

Interventions designed to reduce the use of seclusions/restraints in child and adolescent psychiatric inpatient care

*ACT* Acceptance and Commitment Therapy, *ASD-CP* Autism Spectrum Disorder Care Pathway, *BMP* Behavior Modification Program, *CFCC* Child- and Family-Centered Care, *CPS* Collaborative & Proactive Solutions, *DBT* Dialectic Behavior Therapy, *M-PBIS* A modified version of Positive Behavioral Interventions and Supports, *MBSR* Mindfulness-Based Stress Reduction Training, *Monthly restraint rate per 1000 client days*, Total monthly restraint events, divided by total monthly client days, multiplied by 1000, *PRN* Pro re nata or as needed medication, *S/R* Seclusions/restraints, *S/R/H* Seclusions/restraints/physical holds, *Sensory room*, A specialized room where sensory equipment can be used to help promote emotion regulation, *Six Core Strategies*, 6CS-National Association of State Mental Health Program Directors

### Trauma-Informed Care

The concept of Trauma-Informed Care (TIC) emerged as an effort to address and increase the understanding of trauma, and the importance of identifying it early, in multiple systems of care [50]. Trauma-informed care seeks to understand the connection between presenting symptoms/behaviors and the individual’s trauma history [50]. TIC focuses on doing no further harm or reactivating past traumatic experiences, but instead on promoting healing and growth [50]. Trauma-informed care should be distinguished from trauma-specific treatments (like Trauma-Focused Cognitive Behavioral Therapy) and is a much broader concept, aimed at transforming entire systems of care by embedding an understanding of traumatic responses at all levels. Trauma-informed care should be compassionate, non-coercive, nonviolent, learning, and collaborative [13, 50]. Clients need to feel connected, valued, informed, and hopeful of recovery. The connection between trauma and psychopathology is understood by all staff, and staff work in mindful and empowering ways with individuals, families, and social services to promote and protect the autonomy of the individual [17].

In the U.S., following national media reports in 1998 on deaths and abuses related to coercive interventions, efforts started to reduce the use of seclusions and restraints [51]. The National Association of State Mental Health Program Directors (NASMHPD) developed the Six Core Strategies, an evidence-based practice designed to prevent conflict and violence and the use of seclusion and restraint [17, 32, 51]. The Six Core Strategies are based on child-centered, trauma-informed, and strength-based care, focusing on primary prevention principles [32, 36]. They include: 1) active leadership towards organizational change, e.g. engagement in trauma-informed principles, 2) use of data to inform practice, e.g. data collection of seclusions and restraints, 3) workforce development, e.g. trauma-informed education of staff, 4) use of restraint/seclusion prevention tools, 5) involvement and inclusion of consumers at all levels of care, and 6) debriefing techniques [17, 32]. The Six Core Strategies have been taught throughout the U.S., showing profound results on the use of restraints/seclusion, and are also spreading internationally, adult units included [51].

### Outcome

In this review, four papers (but three facilities) described multi-component, trauma-informed initiatives [32, 33, 36, 37], of which three papers referred to the Six Core Strategies described in the introduction [32, 33, 36]. Azeem et al. (2015) [33] reported a 100% decrease in mechanical restraints in a 52-bed U.S. pediatric psychiatric hospital during a 10-years period and an 88% decrease in physical restraints (statistical significance not mentioned). This facility was also evaluated in a previous article by Azeem et al. 2011 (Table 1) [32]. These articles could serve as an example of the broad variety of interventions that could be included in a trauma-informed and strength-based multi-component initiative, aligning with the Six Core Strategies.

Azeem et al. described how the leadership on all levels supported restraint reduction. The hospital encouraged the use of verbal de-escalation techniques and a focus on primary prevention. The topic of restraint reduction and prevention was a standing agenda item in executive meetings. Every restraint was reviewed on the day following the event. Use of the mechanical restraint bed was prohibited unless both the physician and the supervising nurse were present at the unit. The unit collected data, such as the number of restraints per month, average time in restraints, number of youths involved in these interventions, injuries to staff/youth, etc. These data were shared with staff and leadership. All staff were offered education on trauma-informed care. Training initiatives were also undertaken for staff to learn and implement the principles of DBT, Attachment Regulation and Competency (ARC) framework, and Trauma-Focused Cognitive Behavioral Therapy.

A team was set up to immediately support staff who were assaulted or traumatized. There were also regular team building activities, health education opportunities, and yoga groups to support staff. Families were welcome to visit the hospital before the arrival of a youth to tour the facility. Individualized treatment plans were drawn up with individualized goals, triggers, early warning signs, coping tools, safety plans, interests/hobbies, skills, etc. Staff were informed about the youth’s trauma history and family dynamics. An occupational therapy consultant was utilized to assess the sensory needs of the youths, offering appropriate tools, like sensory brushes or weighted blankets. The unit offered sports and recreational activities like music therapy, art or cooking, and staff worked closely with the youths to build skills. A special team was set up with members particularly skilled at conflict resolution and de-escalation techniques, to be dispatched when necessary. The unit implemented debriefing both immediately after a restraint event and in complex situations again after a few days, for problem solving and planning. The hospital also implemented different youth and family activities to enhance their participation at the facility [33].

In the other trauma-informed initiatives, statistically significant reduction in seclusions and restraints was reported by Wisdom et al. (2015) [36] in three New York-based facilities (patients up to 17 years old), after implementation of the Six Core Strategies (Table 1). Regan et al. (2017) [37] described the implementation of Child and Family Centered Care (CFCC) in a U.S. inpatient unit for children 2–12 years old, where a trauma-sensitive perspective was a part of the initiative, as well as CPS (see below).

### CPS and CFCC

CPS is a psychosocial treatment model for behaviorally challenging youths, based on the assumption that “children do well if they can” [52]. The method was developed by Ross Greene, PhD, and conceptualizes challenging behavior as the byproduct of lagging cognitive skills, particularly in the domains of flexibility/adaptability, frustration tolerance, and problem solving [21, 52]. In CPS, children’s challenging behaviors are said to appear when the expectations placed on the child outstrip the child’s skills [52]. Behavior - whether crying, withdrawing, screaming, hitting, biting - is simply the means by which a child communicates that there is incompatibility between expectations and skills. The model involves engaging caregivers in the process of identifying a child’s lagging skills and unsolved problems, and helping caregivers and youths solve those problems collaboratively and proactively [52].

According to Regan et al. [37], CFCC is a philosophy of care, based on treating people with dignity and respect, sharing information with patients/families, supporting them to build on their strengths and independence and to collaborate in policy/program development. CFCC recognizes that parents themselves need emotional support and acknowledges that a child’s hospitalization is a stressful event in the life of a family. The development of trust is a primary component, and the re-traumatizing potential of procedures must be considered. Evoking feelings of victimization and powerlessness runs counter to the goals of CFCC, which instead promote a nurturing, collaborative approach.

### Outcome

Four units implemented CPS, either as a separate intervention [38], or as part of a multi-component initiative [37, 39, 40]. Ercole-Fricke et al. (2016) [38] evaluated a slightly modified CPS approach in a U.S. adolescent emergency psychiatric inpatient unit, patients 12–17 years old. The 5-year study showed a statistically significant decrease in punitive strategies and techniques, such as a room restriction procedure (Table 1). Restraint episodes were already low according to the authors. and continued to trend downward (not statistically significant).

Sams et al. (2016) [39] evaluated CPS as a part of a multi-component initiative towards strength-based care in a U.S. child and adolescent inpatient unit, patients 5–18 years old. According to the authors, strength-based care is founded on the premise that a person’s skills, interests, and support systems are vital to developing an effective treatment plan. Implementing CPS and strength-based care led to a 75% reduction in total hours of seclusion and restraint over the course of 1 year (statistical significance not mentioned). The initiative also included interventions such as a mindfulness group for adolescents, DBT-group, animal assisted therapy, and acceptance and commitment therapy (ACT). Patients and families reported overall improvement in satisfaction, perception of safety on the unit, helpfulness of group therapy, and involvement in decisions regarding their care. The unit also showed the hospital’s highest staff engagement and satisfaction level.

As noted in the section about Trauma-Informed Care, Regan et al. (2017) [37] included CPS in their CFCC initiative. After the implementation, the unit reported no mechanical restraints since 2001, no locked-door seclusions or chemical restraints since 2002, marked reduction in physical holds (under 5 min) from over 100 per month to less than 10 per month, significant decrease in staff and patient injuries (*p* value not mentioned), reduced staff turnover, and increased job satisfaction.

Weldon Bonnell et al. (2014) [40] studied an inpatient psychiatric facility in Canada, patients’ mean age 14–15 years. External reviewers had assessed the unit due to marked escalation in physical aggression, constant observation, security, and staff sick leave. As a consequence of this, the unit implemented CPS, together with debriefing sessions, representation of multidisciplinary team members, and restructuring of unit staffing. The study reported statistically significant decrease in constant observation each month. There was also a decrease in incidents (verbal or physical aggression, errors by staff and accidents), sick leave, security, and seclusion, but these results were not statistically significant. How CPS was implemented is not outlined in the article, except from staff learning and practicing the model.

### Behavioral Management Programs

Behavioral management is a general category of interventions, grounded in social learning theory and applied behavior analysis. According to social learning theory, people learn within a social context, primarily by observing and imitating the actions of others. Learning is also influenced by being rewarded or punished for particular behaviors. The general aim is to reduce the child’s expression of problem behavior, increase the expression of prosocial behavior, increase the ability to relate to others, and thereby increase overall wellbeing [53].

However, point and level systems, which are integral parts of behavioral modification programs, have been criticized for being counterproductive and sometimes even destructive or punitive in psychiatric milieus, and not considering the child’s capacity to exhibit certain behaviors [54]. Some articles in the review describe going from a behavior management program to another intervention [37–39]. These authors express that the traditional behavioral approach with rewards and consequences based on patients’ behavior often led to increased conflicts between patients and staff [39] or frustration and exacerbated episodes of acting-out behavior [38] and that the staff was seen as “rule-enforcers” and “point-tallyers”, feeling undervalued and unappreciated [37].

There is no universal ‘BMP’ – different behavioral programs include different components or treatment ingredients. As mentioned in the introduction, a previous study reported significant restraint reduction following the implementation of a comprehensive behavioral management program in a child and adolescent inpatient unit [23].

Three types of Behavioral Management were used in three different reviewed articles: Modified Positive Behavioral Interventions and Supports (M-PBIS) [41], Behavior Modification Program (BMP) [42], and “behavioral modification plans” [43]:

1. The M-PBIS involved a three-tier approach: 1) Universal interventions, such as establishing staff commitment, a defined set of positively worded expectations and descriptions of how to meet these expectations, reward system for positive behavior, 5:1 positive to negative adult-child interaction ratio, feedback to staff. 2) Targeted problem-solving conversations with selected patients. 3) Functional behavior assessments and individualized behavior plans for a small minority of patients who continued to have problematic behavior after problem-solving conversations [41]
2. The BMP was modeled for children with attention deficit hyperactivity disorder (ADHD) and oppositional defiant disorder (ODD), and included a points system, social reinforcement, time-out, and parent training [42].
3. The behavioral modification plans addressed issues like problem behavior, replacement behavior, positive rewards, and negative consequences [43].

### Outcome

Reynolds et al. (2016) [41] studied the use of M-PBIS in a psychiatric inpatient unit for high-risk youths. There was a significant decrease in mean seclusion rate, percentage of patients placed in seclusion or restraint, mean duration of seclusion or restraint, and percentage of patients who received any PRN, i.e. pro re nata or as needed medications. (Table 1).

Carlson et al. (2020) [42] studied the effects of discontinuing the use of a BMP in an inpatient unit for children aged 5–12. The authors reported that the subsequent intervention after discontinuation of the BMP focused on talking the child down or distracting him/her from intensely emotional situations so that restrictive interventions would not be needed. Coping skills were still taught and the points system remained but no limits were set, and the new intervention did not provide a comprehensive treatment approach with an evidence-based theoretical framework. Five cohorts of children were studied over a 10-years period. Significantly more children had events of seclusions/restraints/physical holds when BMP was absent. Agitation (measured by PRN use) increased after the discontinuation of BMP. (Table 1) The authors mention that during the BMP-present period, time-outs and “the quiet room” (open-door seclusion) were used, neither of which counted as a seclusion. This might have introduced some degree of bias, artificially reducing the use of seclusions/restraints/physical holds during the BMP-present period.

Eblin (2019) [43] evaluated the implementation of a decision-making algorithm for initiation of seclusions or restraints, behavioral modification plans for patients at risk for seclusions or restraints, and a patient-debriefing tool to be used after a restrictive episode in an inpatient behavioral health unit for children aged 6–17. Post-implementation, data was collected over a 3-month period and showed a 55% decrease in the total seclusion and restraint rate compared to preintervention data (statistical significance not mentioned). The data also showed an overall 29% decline in total time spent in seclusion and restraint, per episode. (Table 1).

### Smaller Interventions (Sensory Rooms, MBSR, Milieu Nurses)

#### Sensory Rooms

A sensory room, sometimes called comfort room or Snoezelen, is a space that contains a variety of tools used to stimulate the senses, e.g. fidget tools, weighted blankets, colored lights, or relaxing music [44]. The aim of a sensory room is to help patients self-regulate, particularly at times of escalation in anxiety and/or aggression, and to offer a safe space for a distressed patient to decompress while preserving autonomy [44]. Three studies in the review evaluated sensory rooms as a stand-alone intervention [44–46]. Sensory rooms or sensory tools provided by an occupational therapist were also included in some trauma-informed, multi-component initiatives [32, 33, 36]. In the three studies evaluating sensory rooms only, the implementation included educating and training staff in the theory behind sensory modulation, how to conduct a sensory room session, and how to decide which patients might need it and also remain safe in the room [44–46]. One session was designed as a 1:1 patient-staff intervention.

#### Outcome

Seckman et al. (2017) [44] studied a U.S. inpatient unit for adolescents aged 12–17 with emotional and behavioral disorders, measuring seclusion/restraint rate 6 months pre/post sensory room initiation. Statistical significance was not reported, but the results suggested a reduction in the incidents of restraint (26.5%) and seclusion (32.8%) and reduced frequency of patient aggression (Table 1). However, the duration of seclusions and restraints increased, something the authors suggested might be attributed to a few patients being “high users”.

West et al. (2017) [45] studied an Australian inpatient unit for adolescents 12–18 years old, comparing 56 sensory room users and 56 non-users. The results showed statistically significant distress reduction following sensory room use and the greatest reduction among adolescents with a history of aggression. There was no significant difference in seclusion rates. Female gender and having an anxiety disorder were associated with sensory room use.

Bobier et al. (2015) [46] studied a child and adolescent inpatient unit in New Zealand, with patients up to 18 years old. The study was a pilot investigation introducing a sensory room in the unit. Patients used the room for both activation and deactivation, with statistically significant effect. There was a statistically significant decrease in seclusion episodes 6 months before vs 6 months after the introduction of sensory rooms, and a slight decrease in full restraint episodes, but not statistically significant. There was a statistically significant increase in partial restraints. (The authors’ definition of partial vs full restraint was not included in the article.)

#### Mindfulness Based Stress Reduction

In a review of ecological factors affecting inpatient psychiatric unit violence, Hamrin et al. (2009) described how violence on an inpatient unit resulted from a multitude of factors, including complex interactions among patients, staff, and the culture of the specific unit [55]. Staff who demonstrated compassionate attitudes toward patients, gave of themselves, showed empathy for patients’ suffering, related authentically to patients, and treated them with dignity and respect had fewer violent encounters [55]. In addition, nurses who were attuned to patient’s fears and interactions with other people had an opportunity for early intervention if they observed intrusive and threatening behavior, and could respond in a violence-lessening way to patients [55]. Thus, it is likely that interventions that enhance the staffs’ listening skills and presence are important in creating a safer environment for patients and staff [47]. Being truly present with patients has also been reported to improve staff’s ability to provide good care [56].

Mindfulness Based Stress Reduction (MBSR) is a structured therapy package, rooted in Theravada Buddhism and westernized by Jon Kabat-Zinn at the University of Massachusetts Medical Center in 1979 [56]. MBSR combines mindfulness-based meditation and Hatha yoga, and includes exercises like body scanning, meditation, and gentle yoga postures. According to a literature review by Praissman (2006) focusing on adults, MBSR is an effective treatment for reducing stress and anxiety for patients, as well as healthcare providers [56].

#### Outcome

In the present review, Hallman et al. (2014) [47] evaluated the implementation of a brief MBSR training program, 8 days instead of the traditional 8 weeks, for interprofessional staff in a U.S. child and adolescent psychiatric acute care unit. The aim was stress reduction and increased unit safety. Two months after the MBSR training period, there was statistically significant stress reduction and increased mindfulness among staff. The increase in mindfulness was seen immediately after the 8-day training period. There was also a positive trend towards increased patient and staff safety with a decreased number of staff call-ins, decreased need for 1:1 staffing episodes, and decreased restraint use 2 onths after the training period (significance not reported). (Table 1).

#### Milieu Nurses

Creating clearer staff roles can have a positive influence on psychiatric violence [55]. Rae Magnowski and Cleveland (2020) [48] described how an inpatient child and adolescent psychiatric unit, patients 5–18 years old, with no additional resources or costs, managed to reduce monthly restraint rates through the implementation of “milieu nurse-client shift assignments”, compared with individual nurse-client shift assignments. The intervention was an innovative change in nurse staffing structures. Instead of dividing up clients and tasks equally among all nurses without regard to client acuity, the nurses were assigned the role as a “task nurse” or a “milieu nurse”. The “task nurse” was responsible for administering scheduled medications and conducting client check ins/safety checks, while the two “milieu nurses” were responsible for providing an environment of structure, safety, consistency and empathy, as well as administering PRN medications and executing/updating client treatment plans.

#### Outcome

The milieu nurse-client shift assignment enabled early intervention and de-escalation of situations with clients who displayed aggressive behaviors. The individual nurse-client shift assignments (the control variable) focused on meeting individual client needs, whereas the milieu nurses focused on the needs of the group as a whole. There was a statistically significant decrease in average monthly restraint rate during the intervention, from 72.9 to 7.5 restraints per 1000 client days [48].

### Autism Spectrum Disorder Care Pathway

According to Kuriakose et al. [35], the Autism Spectrum Disorder Care Pathway (ASD-CP) was developed as an approach to improving care in psychiatric units through autism-specific intervention strategies. Individuals with autism may have limited ability to communicate their symptoms, due to difficulties with social communication or intellectual deficits. They are also typically hospitalized for externalizing problem behavior, such as aggression or self-injury. Without sufficient understanding and clinical experience of autism in psychiatric units, this can lead to an increased risk of harm for both patients and staff. This in turn can increase the risk of inappropriate crisis intervention, like seclusions, restraints, or PRN medication administration [35].

The ASD-CP in the study by Kuriakose et al. emphasized input from caregivers regarding communication skills, early warning signs of agitation, and activity/item preferences. It also included implementing a structured schedule with extensive use of visual supports, teaching patient coping skills, and training staff in the features of ASD [35].

### Outcome

Kuriakose et al. (2018) implemented the ASD-CP in a public hospital child psychiatric service, patients 4–17 years old [35]. The proportion of children experiencing a hold/restraint was significantly smaller after the ASD-CP initiation. The number of holds/restraints, intramuscular medication administrations delivered, and the length of stay trended downward (not significant). These results were described as clinically relevant. The stability of the results was later evaluated by Cervantes et al. (2019) [34]. (Table 1).

### Dialectical Behavior Therapy

Dialectical Behavior Therapy (DBT) is an evidence-based treatment that directly addresses suicidal behavior and self-injury [49]. Tebbett-Mock et al. (2020) [49] evaluated DBT versus treatment as usual (TAU) in a U.S. acute-care psychiatric inpatient unit for adolescents (age 12–17). The DBT milieu treatment in the study included: DBT coaching, a token economy including an egregious (outside limits of the unit) behavior protocol requiring chain and solution analyses for egregious behaviors on the unit, therapeutic environment, resources for use of coping skills, DBT vocabulary, DBT skills groups (focusing on Mindfulness, Distress Tolerance, Emotion Regulation, Interpersonal Effectiveness and Middle Path), additional therapeutic and leisure groups (e.g. pottery making or pet therapy), and intensive psychotherapy, including both individual and family/collateral therapy sessions.

TAU consisted of milieu treatment comprising a token economy system, cognitive-behavioral therapy, skills groups, activity groups focused on general coping skills and mental health wellness, and intensive individual and family/collateral psychotherapy. Treatment provided was cognitive-behavioral therapy, family systems, psychoeducational, and/or supportive in nature [49].

### Outcome

Patients who received DBT had significantly fewer constant observation hours for self-injury, incidents of suicide attempts and self-injury, restraints, and days hospitalized compared to patients who received TAU. No statistically significant differences were found for seclusions or constant observation hours for aggression, incidents of aggression toward patients or staff, or readmissions [49].

## Discussion

Coercive measures in psychiatric care are controversial and have the potential to cause injuries, feelings of victimization, re-traumatization, and PTSD [6, 10]. It is therefore paramount that psychiatric units implement preventive measures. The present review of 18 papers published in the recent decade identified a number of methods used in child and adolescent psychiatric inpatient care to reduce the use of coercive measures. Although a variety of theoretical backgrounds were seen, common mediating factors could be observed in many of the studies.

First, on an organizational level, the units agreed upon a specific method and implemented it systematically. The management and staff shared the same aim, ethical and theoretical framework, and most of the studies described a positive attitude towards change and learning new skills. Education of staff ensured that they had the competence needed for their work. All studies collected data, but only two methods involved sharing data regularly with staff to motivate change [32, 36, 41]. One article provided a cost-savings analysis [49] and some studies briefly mentioned the program costs, e.g. “compensation for a 50% psychologist” [41] or “no additional resources” [48], but most of the studies did not provide a cost analysis.

Second, at a clinical level, all methods included preventive efforts, e.g. early interventions, de-escalation techniques or safety plans. Most interventions were aimed at improving communication skills and affect regulation [32, 33, 35–47, 49], several encouraged patients and parents to be involved in the care [33, 35–37, 39, 44–46], some included debriefing after a coercive intervention [32, 33, 36, 43] or interventions promoting the wellbeing of staff [33, 39, 47, 49]. Behavior was often viewed as a consequence of some factor, e.g. trauma (TIC), lagging skills (CPS), difficulties with emotion regulation (DBT) or autism (ASD-CP). Although all methods aimed to reduce the use of coercive measures, many studies had other variables as their primary target, e.g. suicidal ideation [49], distress reduction [45–47], number of PRNs given for agitation [42], or improved care for children with autism [35]. The Six Core Strategies was presumably the method with the most direct and broad focus on restraint reduction, but the studies on CPS, CFCC, M-PBIS and “the milieu nurse client-shift assignment” also aimed directly at reducing the use of coercive measures.

The material could be divided into six groups or themes: TIC, CFCC and CPS, BMP, smaller interventions, ASD-CP, and DBT. The purpose was to provide insight into the different theoretical backgrounds. The distinction between the first two groups was whether trauma was an essential part of the method or not. Apart from that, the interventions in both groups were multi-modal and shared core values, e.g. collaboration and a compassionate, child-centered care. The Six Core Strategies and CPS were evidence-based methods, whereas TIC, CFCC, and strength-based care were more frameworks or philosophies of care. The third group consisted of methods building on behavioral management, but the M-PBIS was the only full BMP being implemented and evaluated systematically. BMPs in general and the “point and level system” in particular, have been problematized for not taking patients’ skills at performing certain behaviors into account, potentially resulting in a negative dynamic between staff and patient [54]. The M-PBIS included several ingredients that possibly reduced the risk of these negative side-effects, such as a 5:1 positive to negative adult-to-child interaction ratio, regular feedback to staff, weekly staff training sessions, and individualized plans for certain individuals [41]. The fourth group consisted of smaller interventions: MBSR aiming at reducing perceived levels of staff stress by an increase in mindfulness, sensory rooms aiming at reducing patient distress, and the milieu nurse-client shift assignment attempting to reduce restraint rates through a change in nurse staffing structures. The last two groups were more specific to the needs of certain groups of patients: the ASD-CP seeking to improve care for patients with autism in psychiatric units and the DBT primarily focusing on decreasing suicidal ideation, self-injury, and aggression.

Comparing the efficacy of the interventions is challenging, due to the heterogeneity of study designs and outcome measures. It is also difficult to determine whether some components in the methods were more critical to the outcome than others. The mere fact that a unit put restraint reduction on the agenda and agreed upon a method could have an impact. With that said, it appears as if the larger collaborative and trauma-informed interventions like the Six Core Strategies [32, 33, 36] and CPS in combination with either CFCC or strength-based care [37, 39] showed the most prominent results. Interestingly, these studies reported the strongest support from their leadership and had restraint reduction as a primary target. These methods also acknowledged psychiatric inpatient violence as a complex phenomenon that must be viewed in a context, as a result of interactions among patients, staff, and the unit culture [55]. Apart from reducing the use of coercive measures, the methods increased job satisfaction [37, 39], reduced staff turnover [37, 38], increased satisfaction among patients and families, and led to an increase in overall creativity in the unit [39]. Two of these initiatives [33, 37] had been in place for many years and reported a complete and lasting elimination of mechanical restraints, which is often seen as the most distressing coercive measure. However, most studies reported a reduction in the use of seclusion or restraint and the methods could be considered, depending on the needs of the unit [35, 38, 40–43, 47, 49]. The only exception were the studies on sensory rooms, which showed mixed results [44–46].

For a unit with strong resources seeking to reduce the use of coercive measures, all interventions in group 1–2 seem to be good options, e.g. TIC, Six Core Strategies, strength-based care, CFCC, and CPS. Since the Six Core Strategies and CPS were the only evidence-based methods while the others represented philosophies of care, they are probably preferable and easier to implement. Behavioral management also seems to be effective in reducing the use of coercive measures, and for units preferring this, M-PBIS could be an option. For units with a smaller budget, the “milieu nurse-client shift assignment” exemplifies an inexpensive intervention, which also seemed to be effective and could be a candidate for further studies [48].

Six Core Strategies, CPS and behavioral management have also been evaluated in previous research. CPS resulted in a 99% reduction in the number of restraint episodes in a psychiatric unit for children aged 3–14 [21], and a significant reduction in restraints (97%, *p* < .001) and seclusions (69%, *p* < .001) in a psychiatric unit for children aged 4–12 [22]. CPS has been shown to be effective in many different settings, summarized in a review by Greene et al. [52], including one randomized control trial examining the efficacy of CPS in treating patients with ODD [57]. The Six Core Strategies have shown statistically significant reduction in the use of restraint and seclusion in the U.S. and internationally, adult units included [51, 58, 59]. Finally, a behavioral management program implemented in a psychiatric facility for children and adolescents age 4–18 resulted in a significant decrease in the number of restraints [23].

Implementing new interventions can be a challenge, calling for an extended and inclusive decision-making process based on principles of a learning organization, shared visions, and team learning to make it possible [60]. Processes like these are often slow and organic, involving many individuals who need to prioritize the same goals and start moving in the same direction. Wisdom et al. noted that the Six Core Strategies were not “golden keys”; successful implementation required major and continuous commitment on all levels, collaboration and a willingness to learn [36]. Similar factors were found to be important when implementing TIC in child and adolescent units, e.g. commitment from leadership, sufficient staff support, family involvement, aligning policies with the principles of TIC, and continuous evaluations to motivate change [61].

Several of the findings in the present review could also be put in the context of salutogenesis. Antonovsky, the founder of the theory, argued that pervasive stressors are inevitable in life, and what promotes health is our ability to experience life as comprehensive, manageable and meaningful, the cornerstones of his concept Sense of Coherence (SOC) [62]. Adolescents admitted to psychiatric inpatient care are often traumatized, come from poor socioeconomic backgrounds, and describe a dysfunctional family situation indicating a weak SOC.

We believe that many of the methods in the present review can have a positive impact on the patients’ SOC. TIC strives to increase their understanding of the connection between past trauma and current symptoms. CPS helps the patient to feel understood in challenging situations. BMP places emphasis on social learning and behavioral analysis. These methods are likely to strengthen the comprehensive component of SOC. TIC also teaches conflict resolution and de-escalation techniques. In CPS, cognitive skills and problem-solving techniques are taught, in BMP different reward systems are practiced, increasing the manageability component of SOC. Several of the reported methods in the present review e.g. CPS, CFCC, TIC and MBSR, may also strengthen the staff’s experience of comprehensibility and manageability. Linked to the comprehensibility and manageability components of SOC is the concept of locus of control (LOC). Feeling control over one’s life events is referred to as internal LOC; conversely, when feeling that chance, luck, fate, or powerful others are in charge, LOC is considered more external [63]. Psychiatric symptoms and adverse life-events are associated with external LOC [64] while patients with lower levels of psychiatric symptoms present more internal LOC [62]. Several of the measures in the present review, e.g. TIC, CPS, DBT and BMP, focus on manageable problem-solving methods that can probably increase the patient’s internal LOC. Through increased SOC and LOC among both patients and staff, conditions are likely to be created for a care environment with fewer coercive measures.

There was a clear dominance of articles of U.S. origin in this review. Some studies from other countries, such as Norway [25, 26], Finland [9, 30] and Israel [12], covered other aspects of the subject, e.g. prevalence, characteristics of patients frequently restrained, or patients’ attitudes towards restrictive measures, but none of these studies met our inclusion criteria. We also excluded non-English language articles. An assumption could be that the issue of restraint has been of greater concern in the U.S. Even though comparing practices between countries is challenging, the lack of studies from other parts of the world is difficult to explain, and more research in this field has been called for [20, 24, 65].

Psychiatric care in child and adolescent inpatient units should always strive to be as respectful and empowering as possible, maintaining a safe and trustful environment, while respecting the child’s integrity. This implies keeping interventions that have the power to leave patients feeling shameful, angry, or victimized to a minimum. However, there might always be situations when a restrictive intervention is unavoidable, and the only way to protect a child or adolescent in a psychiatric unit from hurting him/herself or others. Considering the possible negative consequences of such interventions, they can only be ethically defendable if psychiatric organizations work continuously and systematically to prevent them. This review shows that effective and lasting restraint reduction is possible and that it can be combined with and achieved through a child-centered, compassionate, and empowering psychiatric care.

## Limitations

There was a wide range of study designs in the present review, and four papers were of a more descriptive character. Some authors labelled the method and the implementation process in detail, others more briefly. There was a lack of controls and not necessarily causal effect. The intervention components that were most instrumental in achieving outcomes could not be identified. Because of the design heterogeneity we did not use a scoring system to evaluate the quality of the included studies, which is a limitation. The findings should be seen as trends rather than generalizable results, due to the different quality of the studies and various age spans and characteristics of the patients. A majority of the articles were of U.S. origin, which might affect the generalizability. The generalizability was also challenged by the lack of standards when it comes to the definition of restraint and how to report the results.

## Strengths

The present review is based on a systematic search identifying the most recent studies on how to reduce the use of coercive measures in child and adolescent psychiatric inpatient care, involving 18 studies published in the last decade. To our knowledge, the last review in the field covered three articles published 2006–2013 [20]. Since this is a subject where important research might be done in the form of smaller studies or quality improvement projects, our decision to include all types of study designs adds to previous reviews with an updated and broad description of the field.

## Research Implications

This article shows that reducing the use of restraints and seclusions is possible and should be prioritized. To facilitate comparisons, future models should evaluate different standardized parameters, including patient satisfaction.

## Data Availability

Not applicable.

## Abbreviations

*ACT*: Acceptance and Commitment Therapy
*ASD-CP*: Autism Spectrum Disorder Care Pathway
*BMP*: Behavior Modification Program
*CBM*: Comprehensive Behavioral Management Program
*CFCC*: Child- and Family-Centered Care
*CPS*: Collaborative & Proactive Solutions
*DBT*: Dialectic Behavior Therapy
*M-PBIS*: A modified version of Positive Behavioral Interventions and Supports
*MBSR*: Mindfulness-Based Stress Reduction Training
*PRN*: Pro re nata or as needed medication
*TAU*: Treatment as Usual
*TIC*: Trauma-Informed Care
